# STAT6-positive solitary fibrous tumour of the lacrimal gland with floret-type giant cells: a rare case report

**DOI:** 10.1186/s12886-026-05525-x

**Published:** 2026-11-05

**Authors:** Alaa Sonbol, Raghda Tarek, Maha Elzaafarany, Rahma Abdelaziz, ZM Elbeah, Rami Khader, Mohammed Bdair, Lama Nazzal, Esraa Elhakim

**Affiliations:** 1 Medical Oncology Unit, Oncology Center, Mansoura University, Mansoura, Egypt; 2 Department of General Surgery, NMC Healthcare Ltd, Abu Dhabi, United Arab Emirates; 3 Faculty of Medicine, Mansoura University, Mansoura, Egypt; 4 Department of Medicine, Faculty of Medicine and Health Sciences, An-Najah National University, Nablus, Palestine; 5 Faculty of Medicine, Arab American University, Jenin, Palestine

**Keywords:** Solitary fibrous tumour, Lacrimal gland, STAT6, Floret-type giant cells, Spindle-cell tumour, Orbit

## Abstract

**Background:**

Solitary fibrous tumours (SFTs) are rare fibroblastic neoplasms, and involvement of the lacrimal gland is uncommon.

**Case presentation:**

A non-smoking Egyptian man aged 52 years presented with a five-month history of enlarging left lacrimal swelling, proptosis, and mildly reduced left visual acuity (6/9 at presentation). Non-contrast orbital computed tomography showed a well-defined 9 × 12 mm lesion without bone erosion or local invasion. Complete surgical excision was performed. Histopathological examination demonstrated a patternless spindle-cell tumour with staghorn vasculature, low mitotic activity, and floret-type multinucleated giant cells, without necrosis, lymphovascular invasion, or perineural invasion. The tumour cells expressed CD34, CD99, BCL2, and diffuse nuclear STAT6, supporting the diagnosis of SFT. Surgical margins were negative. At three-month follow-up, visual function had fully recovered, with no diplopia.

**Conclusions:**

This report adds a small, completely excised, low-risk example of lacrimal gland SFT in which floret-type giant-cell morphology is documented alongside diffuse nuclear STAT6 staining. Recognition of this pattern is important because it may overlap with other giant-cell-rich spindle-cell lesions. Complete excision with negative margins and risk-adapted long-term surveillance remain appropriate management principles.

## Introduction

Solitary fibrous tumours (SFTs) are fibroblastic mesenchymal neoplasms that were first described in the pleura but are now recognised at multiple extrapleural sites [1, 2]. Most SFTs harbour a NAB2–STAT6 fusion, and strong nuclear STAT6 immunoreactivity is widely used as a sensitive diagnostic surrogate that helps distinguish SFT from histological mimics [1–3].

Orbital SFTs are uncommon, and involvement of the lacrimal gland or lacrimal gland fossa is particularly rare [4–6]. These tumours may present as slowly enlarging orbital masses and can clinically overlap with epithelial, lymphoid, inflammatory, and other mesenchymal lesions. Histologically, a subset of SFTs shows giant-cell-rich morphology with floret-type multinucleated giant cells, a pattern historically described within the giant cell angiofibroma spectrum [7–9].

Lacrimal gland SFTs have been reported previously, including conventional STAT6-positive lesions and giant-cell-rich variants [6, 8, 10, 11]. Accordingly, the added value of the present report is not the lacrimal location alone. Rather, it documents a small, completely excised, low-risk tumour in which floret-type giant-cell morphology is demonstrated together with diffuse nuclear STAT6 positivity, and it places these findings in the context of prior lacrimal gland SFT reports.

## Case presentation

A non-smoking Egyptian man aged 52 years presented with a gradually enlarging swelling in the left lacrimal gland region. The swelling had first been noticed approximately five months before presentation and had slowly increased in size. At presentation, left visual acuity was mildly reduced to 6/9, while the right eye remained unaffected. He denied metamorphopsia, visual-field defects, ocular pain, pruritus, discharge, excessive tearing, or symptoms suggestive of ocular infection.

There was no history of contact lens use, ocular trauma, previous ocular surgery, or thyroid disease. His medical history was otherwise unremarkable, with no hypertension or diabetes mellitus. There was no family history of ocular disease or malignancy.

On examination, there was a unilateral swelling in the left lacrimal gland fossa. The mass was firm, mobile, and non-tender, with associated proptosis. There was no overlying erythema, excessive lacrimation, pus regurgitation, or restriction of eyelid movement. Slit-lamp and fundoscopic examinations were unremarkable. Regional head and neck examination showed no thyroid enlargement, lymphadenopathy, or clinical evidence of sinus-related inflammation. Systemic examination was otherwise unremarkable.

### Investigations

#### Laboratory investigations

As part of the routine preoperative work-up, the patient underwent a complete blood count, coagulation profile, liver function tests, renal function tests, and viral screening for hepatitis B virus, hepatitis C virus, and human immunodeficiency virus. Serum creatinine was elevated at presentation, measuring 2 mg/dL, and the patient was referred for nephrology assessment. His creatinine subsequently improved to 1.2 mg/dL. Because of the initial renal impairment, contrast-enhanced imaging was avoided at presentation.

#### Radiological investigations

**Fig. 1:**
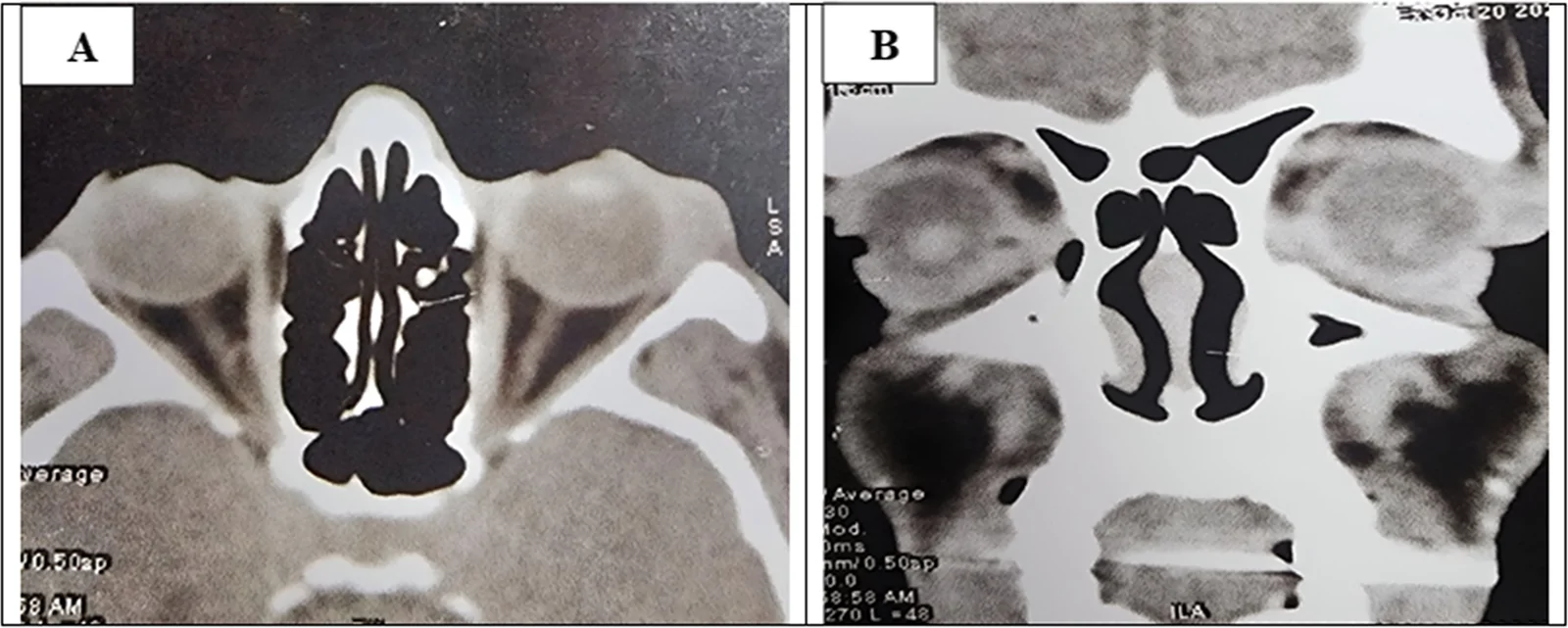
**(A)** NCCT film, axial view, showing a well-defined, isodense, left orbital swelling with no signs of local invasion or bone erosion. **(B)** NCCT film, coronal view, showing a well-defined, isodense, left orbital swelling with no signs of local invasion or bone erosion

A non-contrast computed tomography scan of the orbit was performed in October 2024. The scan showed a well-defined, isodense left orbital lesion measuring approximately 9 × 12 mm in the lacrimal gland region, with associated proptosis. No gross bone erosion, obvious local invasion, or globe abnormality was identified on CT. The preoperative axial and coronal CT images are shown in Fig. 1A and B.

Preoperative MRI was not performed, which limited radiological assessment of soft-tissue extension, orbital apex involvement, perineural spread, dural involvement, bone marrow infiltration, and the lesion’s relationship to the optic nerve and globe.

**Fig. 2:**
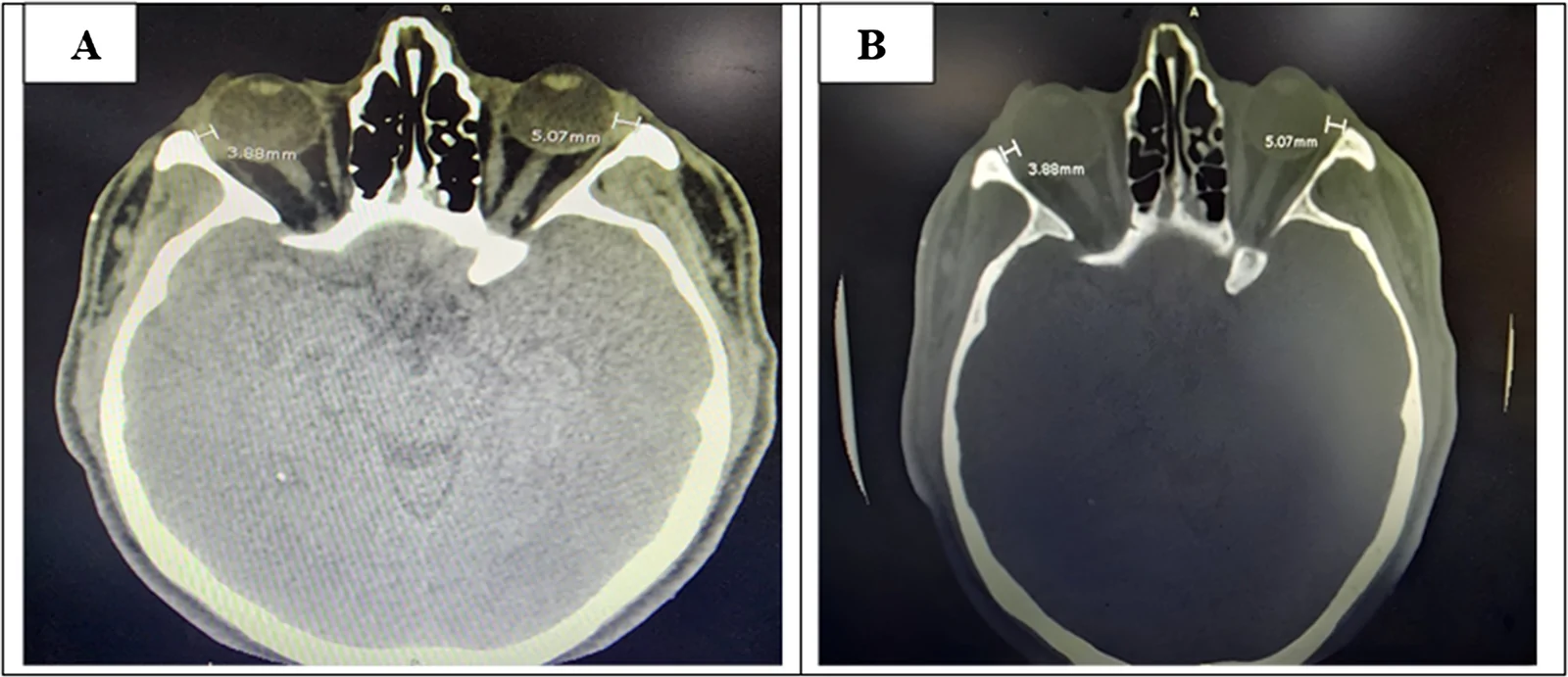
**(A)** Contrast-enhanced CT study, axial view, showing mildly swollen soft tissue in the left orbit, representing postoperative surgical bed changes. **(B)** Contrast-enhanced CT study, axial view, showing mildly swollen soft tissue in the left orbit, representing postoperative surgical bed changes

One month after surgery, once the serum creatinine had improved to 1.2 mg/dL, a contrast-enhanced CT scan was performed. This showed mildly swollen soft tissue in the left orbital operative bed, measuring approximately 5 mm in maximum thickness, which was interpreted as postoperative surgical change. No gross residual lesion was identified on postoperative CT, as shown in Fig. 2A and B.

A contrast-enhanced pan-CT scan was also performed after the diagnosis of lacrimal gland solitary fibrous tumour to screen for similar tumour pathology at other sites. No additional lesions were identified.

#### Histopathological examination

**Fig. 3:**
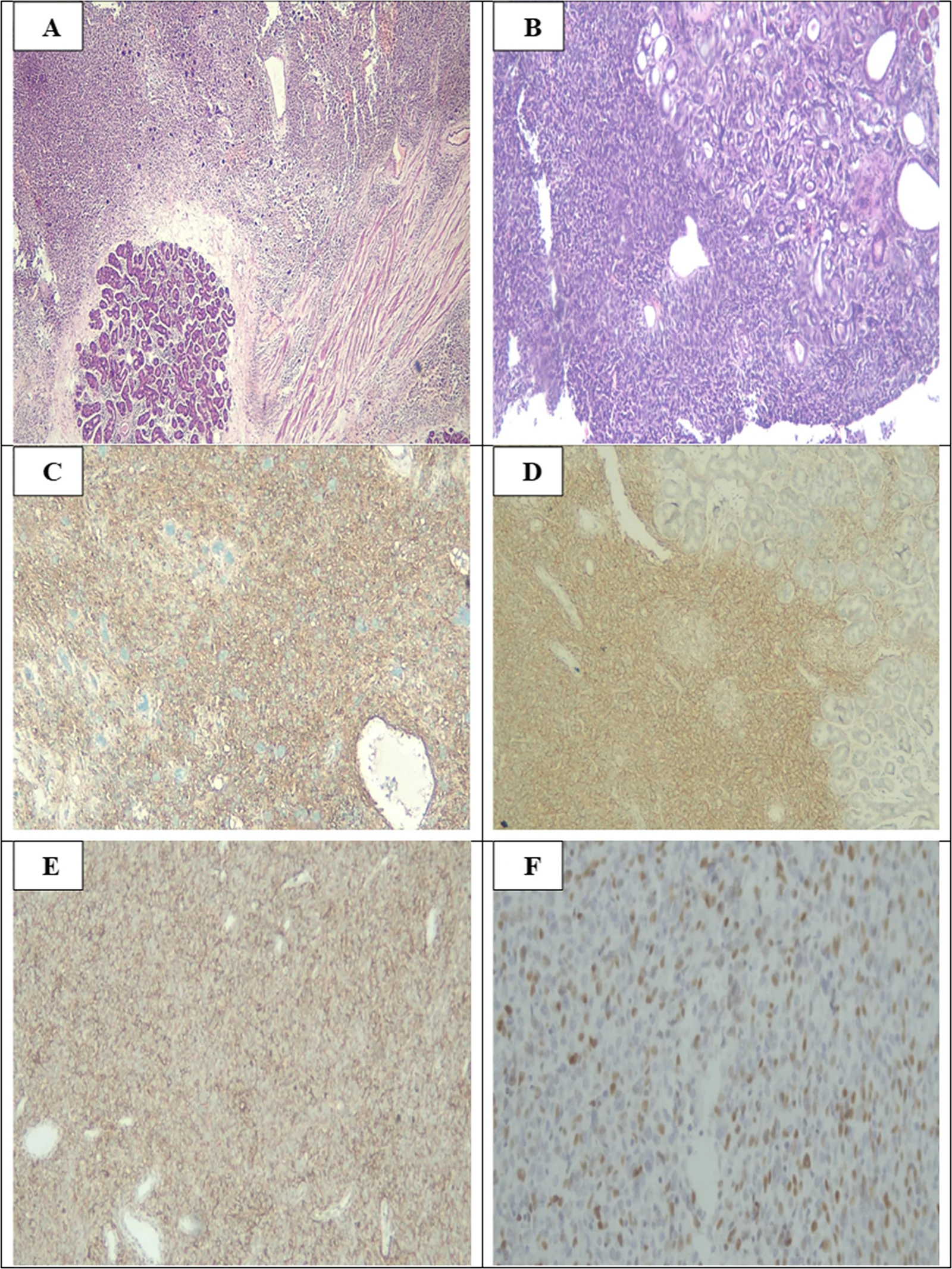
**A**: H&E staining, ×40, showing spindle-cell tumour involving the lacrimal gland and adjacent muscle, with scattered giant cells.** B**: H&E staining, ×100, showing tumour involvement of the lacrimal gland and staghorn-pattern vasculature.** C**: CD34 immunohistochemistry, ×100, showing diffuse strong positivity. **D**: CD99 immunohistochemistry, ×100, showing diffuse positivity. **E**: BCL2 immunohistochemistry, ×100, showing cytoplasmic positivity. **F**: STAT6 immunohistochemistry, ×100, showing diffuse nuclear staining in tumour cells

The patient underwent elective surgical excision of the left lacrimal gland tumour. The surgical specimen was received intact and without fragmentation. Microscopic examination showed a multifocal spindle-cell proliferation involving the lacrimal gland and adjacent muscle tissue, as demonstrated in Fig. 3A.

The tumour showed a patternless architecture with scant stroma, occasional myxoid foci, and peripheral collagen bundles. Frequent branching hemangiopericytoma-like vessels with a staghorn pattern were identified, as shown in Fig. 3B. Floret-type multinucleated giant cells were present, predominantly in loose stromal areas and occasionally near vessel lumens. There was no tumour necrosis, lymphovascular invasion, or perineural invasion. The mitotic count was low, with 1–3 mitotic figures per 10 high-power fields.

#### Immunohistochemistry

Immunohistochemical staining showed diffuse positivity for CD34, CD99, and BCL2 in the spindle tumour cells, as shown in Fig. 3C and D, and Fig. 3E, respectively. The tumour cells were negative for desmin, myogenin, cytokeratin, and CD31. CD68 was negative.

The Ki-67 proliferation index was low, with labelling in approximately 1–2% of cells, mainly among multinucleated giant cells. TLE-1 showed focal nuclear staining in less than 20% of cells and was considered non-supportive of synovial sarcoma.

Because the morphology raised the possibility of solitary fibrous tumour and its histological mimics, STAT6 immunohistochemistry was subsequently performed and showed diffuse nuclear positivity in the tumour cells, supporting the diagnosis of solitary fibrous tumour, as shown in Fig. 3F. Molecular testing for NAB2–STAT6 fusion was not performed. All examined surgical margins were free of tumour cells.

### Diagnostic assessment

The combined morphological and immunohistochemical findings supported a diagnosis of giant cell-rich SFT with floret-type giant cells. The principal histological mimic was deep fibrous histiocytoma; however, the absence of convincing storiform architecture together with the patternless architecture, staghorn vasculature, CD34 positivity, and diffuse nuclear STAT6 staining strongly favoured SFT [12, 13].

### Treatment

The patient underwent elective surgical excision of the lacrimal gland tumour. He recovered uneventfully after surgery and reported gradual improvement of diplopia immediately postoperatively. The decline in visual acuity was followed by ophthalmology and managed with active observation.

Because the lesion was small, low-risk, and completely excised with histologically negative margins, no adjuvant radiotherapy or systemic therapy was administered. The patient was counselled regarding the potential risk of local recurrence, distant metastasis, and rare malignant progression. He was advised to maintain regular outpatient follow-up and to report any new orbital symptoms or new body swellings.

### Outcome and follow-up

The patient was scheduled for regular follow-up every 3–6 months initially. As of 13 May 2025, he had attended one follow-up appointment three months after surgery and was doing well, with full return of visual function, no need for optical correction, and no diplopia.

Postoperative contrast-enhanced CT showed postoperative soft-tissue change in the operative bed and no gross residual disease. However, microscopic completeness of excision was determined by histological margin assessment, which confirmed negative surgical margins.

In view of the absence of preoperative MRI, interval orbital MRI is planned during follow-up, preferably with gadolinium if renal function remains stable, or without contrast if contrast administration is not appropriate.

## Discussion

The present case represents a lacrimal gland SFT with floret-type multinucleated giant cells and diffuse nuclear STAT6 positivity. Lacrimal gland SFT has been reported previously; therefore, the contribution of this report lies in the clinicopathological combination rather than in the anatomical site alone. Specifically, the lesion was small, completely excised, margin-negative and low risk by the Demicco model, yet it displayed the uncommon floret-type giant-cell phenotype together with diffuse nuclear STAT6 staining.

Histologically, the tumour showed patternless spindle-cell growth, staghorn-pattern vasculature, collagenous stroma, low mitotic activity, and no necrosis, lymphovascular invasion, or perineural invasion. Floret-type multinucleated giant cells were present in loose stromal areas and near vessel lumens. CD34, CD99, and BCL2 supported SFT, whereas diffuse nuclear STAT6 was the most diagnostically specific finding in this setting [1–3, 13]. Molecular testing for NAB2–STAT6 fusion was not performed; however, the morphology and diffuse nuclear STAT6 staining were concordant with SFT.

Comparison with prior lacrimal gland/lacrimal fossa reports helps define what this case adds (Table 1). Son et al. described a 39-year-old man with a 1.1-cm lacrimal gland SFT containing peripheral floret-type giant cells and showing CD34 and CD99 positivity [8]. Mupas-Uy et al. reported a 25-year-old woman with a well-circumscribed lacrimal fossa SFT confirmed by STAT6 immunohistochemistry and no recurrence at 6 months [6]. Gheorghisan-Galateanu et al. described a 42-year-old woman with a much larger pseudocystic lacrimal gland SFT showing strong STAT6 and CD34 positivity and no recurrence at 15 months [10]. More recently, Alsaadi et al. reported a 47-year-old man with a 3.8-cm giant cell-rich lacrimal gland SFT with marked angiomatoid/cystic change, diffuse STAT6 expression, and a NAB2ex3–STAT6ex18 fusion; subtotal resection was required because of hypervascularity and intraoperative bleeding [11]. In contrast, the present tumour was only 9 × 12 mm, lacked adverse histological features, and was completely excised with negative margins. This comparison shows that floret-type giant-cell morphology can also be present in a small lesion lacking adverse histological features.

The principal post-excision differential diagnosis was deep fibrous histiocytoma, which may show spindle-cell morphology and multinucleated giant cells but more often demonstrates storiform architecture and lacks diffuse nuclear STAT6 expression [12, 13]. The patternless architecture, staghorn vasculature, CD34 positivity, and diffuse nuclear STAT6 staining in the present tumour strongly favoured SFT. Once these defining findings were established, an extensive tabulation of multiple less likely differential diagnoses was unlikely to add diagnostic value.

Floret-type giant cells are relevant because they place the lesion within the giant-cell-rich SFT spectrum historically associated with giant cell angiofibroma [7, 9]. Their presence may create diagnostic confusion with other giant-cell-rich spindle-cell lesions, particularly when the lesion arises in an uncommon site. The present case therefore reinforces the importance of integrating morphology with STAT6 immunohistochemistry rather than relying on any single microscopic feature.

Complete surgical excision remains the main treatment for localised SFT. In this patient, the tumour was small, completely excised with negative margins, and lacked adverse histological features such as necrosis or high mitotic activity. No adjuvant radiotherapy or systemic therapy was therefore administered. Using the revised four-variable Demicco model, the tumour scored 1 point and fell within the low-risk category [14] (Table 2). Adjuvant treatment is generally reserved for selected recurrent, unresectable, margin-positive, or higher-risk disease [15, 16].

Despite the low-risk classification, long-term surveillance is appropriate because SFTs can recur or metastasise late, including after apparently complete excision [2, 17]. There are no universally accepted surveillance guidelines specific to lacrimal gland SFT, so follow-up should be individualised according to site, margin status, histological features, and patient-specific factors. Interval orbital MRI is planned when clinically appropriate, particularly because preoperative MRI was not obtained.

This report has several limitations. The available postoperative follow-up is limited to three months, preoperative MRI was not performed because of the initial renal impairment, and molecular confirmation of the NAB2–STAT6 fusion was not available. In addition, conclusions from a single case cannot establish tumour behaviour or optimal surveillance intervals. These limitations support cautious interpretation and continued long-term follow-up.

**Table 1:** Selected published lacrimal gland/lacrimal fossa SFT reports compared with the present case

Report	Clinical / imaging features	Pathology / immunohistochemistry	Management / outcome
Son et al., 2013 [8]	39-year-old man; painless left upper-eyelid mass; 1.1-cm well-defined lacrimal gland nodule.	Patternless/focally storiform spindle-cell tumour with peripheral floret-type giant cells; CD34 and CD99 positive.	Mass excision; follow-up duration not reported in the article.
Mupas-Uy et al., 2016 [6]	25-year-old woman; palpable mass and proptosis; well-defined lacrimal fossa lesion without bone invasion.	Spindle-cell SFT with collagenised stroma and staghorn vessels; STAT6, CD34, CD99 and BCL2 positive.	Complete excision; no evidence of recurrence at 6 months.
Gheorghisan-Galateanu et al., 2019 [10]	42-year-old woman; 8-month exophthalmia and epiphora; 4 × 2.2 × 2.7 cm pseudocystic lacrimal gland mass.	SFT with branching vessels; strong diffuse CD34 and STAT6 positivity.	Total tumour excision with dacryoadenectomy; no recurrence at 15 months.
Alsaadi et al., 2022 [11]	47-year-old man; long-standing proptosis; 3.8 × 3.5 × 2.0 cm solid-cystic lacrimal gland mass.	Giant cell-rich SFT with diffuse CD34/STAT6 expression; NAB2ex3–STAT6ex18 fusion and TP53 mutation.	Subtotal resection because of hypervascularity and bleeding; no obvious regrowth at 3 months.
Present case	52-year-old man; 5-month painless lacrimal swelling and proptosis; 9 × 12 mm lesion.	Patternless spindle-cell tumour with staghorn vessels and floret-type giant cells; CD34, CD99, BCL2 and diffuse nuclear STAT6 positive.	Complete excision with negative margins; no gross residual lesion on postoperative CT; clinically well at 3 months.

**Table 2:** Demicco risk stratification applied to the present case

Parameter	Finding in the present case	Score
Age at diagnosis	52 years; below 55 years	0
Tumour size	9 × 12 mm; below 5 cm	0
Mitotic count	1–3 mitoses per 10 high-power fields	1
Tumour necrosis	Absent	0
Total score	Low-risk category	1

Interpretation: Under the revised four-variable Demicco model, a total score of 1 falls within the low-risk category (score 0–3) [14]

## Conclusions

This report adds a small, completely excised, low-risk example of a lacrimal gland SFT with the uncommon floret-type giant-cell phenotype and diffuse nuclear STAT6 positivity. The case illustrates how this morphology can overlap with other giant-cell-rich orbital spindle-cell lesions and how STAT6 immunohistochemistry helps establish the diagnosis. Complete excision with negative margins was appropriate for this low-risk tumour. Because late recurrence or metastasis can occur in SFT, continued risk-adapted long-term surveillance remains warranted.

## Data Availability

All data relevant to this case are included in this article. No additional datasets were generated or analysed during the current study.

## Abbreviations

SFT: Solitary fibrous tumour
CT: Computed tomography
NCCT: Non-contrast computed tomography
MRI: Magnetic resonance imaging
IHC: Immunohistochemistry
STAT6: Signal transducer and activator of transcription 6
NAB2: NGFI-A-binding protein 2
BCL2: B-cell lymphoma 2
CD: Cluster of differentiation
Ki-67: Kiel-67 proliferation index
TLE-1: Transducin-like enhancer of split 1
N/A: Not applicable
